# Sources of Blood Meals of Sylvatic *Triatoma guasayana* near Zurima, Bolivia, Assayed with qPCR and *12S* Cloning

**DOI:** 10.1371/journal.pntd.0003365

**Published:** 2014-12-04

**Authors:** David E. Lucero, Wilma Ribera, Juan Carlos Pizarro, Carlos Plaza, Levi W. Gordon, Reynaldo Peña, Leslie A. Morrissey, Donna M. Rizzo, Lori Stevens

**Affiliations:** 1 Department of Biology, University of Vermont, Burlington, Vermont, United States of America; 2 Vector-borne Diseases Section, Tennessee Department of Health, Nashville, Tennessee, United States of America; 3 Facultad de Bioquímica, Universidad de San Francisco Xavier de Chuquisaca, Sucre, Bolivia; 4 Departamento de Entomología, Servicio Departamental de Salud, Sucre, Bolivia; Mahidol University, Thailand

## Abstract

**Background:**

In this study we compared the utility of two molecular biology techniques, cloning of the mitochondrial *12S* ribosomal RNA gene and hydrolysis probe-based qPCR, to identify blood meal sources of sylvatic Chagas disease insect vectors collected with live-bait mouse traps (also known as Noireau traps). Fourteen *T. guasayana* were collected from six georeferenced trap locations in the Andean highlands of the department of Chuquisaca, Bolivia.

**Methodology/Principal Findings:**

We detected four blood meals sources with the cloning assay: seven samples were positive for human (*Homo sapiens*), five for chicken (*Gallus gallus*) and unicolored blackbird (*Agelasticus cyanopus*), and one for opossum (*Monodelphis domestica*). Using the qPCR assay we detected chicken (13 vectors), and human (14 vectors) blood meals as well as an additional blood meal source, *Canis sp*. (4 vectors).

**Conclusions/Significance:**

We show that cloning of *12S* PCR products, which avoids bias associated with developing primers based on *a priori* knowledge, detected blood meal sources not previously considered and that species-specific qPCR is more sensitive. All samples identified as positive for a specific blood meal source by the cloning assay were also positive by qPCR. However, not all samples positive by qPCR were positive by cloning. We show the power of combining the cloning assay with the highly sensitive hydrolysis probe-based qPCR assay provides a more complete picture of blood meal sources for insect disease vectors.

## Author Summary

The World Health Organization (WHO) estimates that 7 to 8 million people are currently infected with *Trypanosoma cruzi*, the parasite that causes Chagas disease. The WHO recommends insect vector control as the primary prevention method; and insecticide spraying is the most commonly used intervention technique. Sylvatic insect vectors are a special concern because they are a source of reinfestation after insecticides have been applied to living quarters (domestic) and immediate surroundings (peridomestic). To better understand sylvatic insect vector movement, we used two molecular biology techniques to detect the blood meal sources of sylvatic insect vectors. The first technique, cloning of *12S* PCR products, allows us to cast a wide net and detect blood meal sources with no previous knowledge of vertebrates or mammals in the study site. After acquiring knowledge of vertebrates in the study site (either through the aforementioned cloning technique, literature review or survey of the area), the second technique, the species-specific hydrolysis probe-based qPCR provides a highly sensitive assay for particular taxa.

## Introduction

Blood-feeding insects in the subfamily Triatominae are vectors of *Trypanosoma cruzi*, the parasite that causes Chagas disease. Approximately 140 species of Triatominae range across the Americas [1] and vary in their role in transmitting human Chagas disease in part because of their preference for sylvatic (wild), peridomestic (immediate surroundings of a house) or domestic (within house) ecotopes. Although it is generally assumed that domestic and peridomestic vectors are important in disease transmission, the role of sylvatic vectors in disease transmission is less understood. Because sylvatic vectors have the potential to colonize houses or simply enter houses to feed and then leave, collecting sylvatic vectors and analyzing their blood meal profiles can provide insight into their movement among domestic, peridomestic and sylvatic ecotopes and their potential role in disease transmission [2].

Not only vector species, but also vertebrate hosts vary in relevance for human transmission. Vectors that feed on human blood are important in disease transmission but because some mammals are more likely to transmit the parasite to the vectors, insight into the spectrum of blood meal sources is also epidemiologically important. For example, non-infected *Triatoma infestans*, often cited as the most important vector of Chagas disease [3], were 50 times more likely to become infected when feeding on dogs compared to humans [4]. Dogs are also a more dependable food source relative to other hosts, evident by vectors feeding more consistently on dogs across study sites and seasons [5]. A strong correlation between vector parasite infection and vectors feeding on dogs has been reported [6].

Several techniques have documented blood meal profiles of these triatomine insect vectors such as protein-based assays (e.g., antisera and precipitin tests [6]–[8]), DNA tests based on the polymerase chain reaction (PCR, e.g., gel electrophoresis [9], [10], melt curve analysis [11], [12]), direct sequencing [13], [14] and cloning followed by sequencing of PCR products [15]. DNA-based approaches have the advantage of being more amenable to the degraded DNA often found in the vector digestive tract. Except for techniques involving DNA sequencing, these approaches require antibodies, PCR primers, or restriction enzymes designed to detect specific taxa, and the scope is limited by *a priori* knowledge of potential blood meal sources [16]. For domestic and peridomestic vectors, common blood meal sources (e.g., humans, domestic animals) are known [10]–[13], [15]–[18]. However, it is challenging to identify potential sources of blood meals for sylvatic vectors. Amplifying DNA from the blood meal using vertebrate or mammalian specific primers followed by cloning of the PCR products has the advantage of being able to cast a wide net in detecting vector blood meal sources [15]; however, cloning is more costly and time consuming than other DNA-based approaches. Cloning, reliant on conventional PCR may be biased toward more recent blood meals because of vector digestion of older blood meals [16], [19], and thus less sensitive to all blood meal sources, relative to modern highly sensitive DNA-based approaches such as qPCR [16], [20]. The relative sensitivity and specificity of cloning vs. qPCR in detecting blood meal sources collected from the harsh environment of the vector digestive tract has not been examined.

We explore the application of these two molecular techniques to examine the role of sylvatic vectors in the transmission of human Chagas disease, by determining the blood meal sources of sylvatic vectors collected from a region in Bolivia with high disease incidence [21]. We compared two approaches designed to detect blood meal sources from DNA extracted from the vector abdomen. The first approach, cloning of PCR products amplified with general vertebrate primers, broadly identifies all potential vertebrate blood meal sources [15]. The second approach uses highly sensitive qPCR taxa-specific primers and hydrolysis probes to survey for chicken, *Canis sp.* (e.g., dog, wolf and coyote) and human blood meals. To our knowledge this is the first study to use hydrolysis probe-based qPCR to analyze blood meal sources of insect disease vectors and compare the two DNA-based methods for sensitivity and specificity.

## Methods

### Ethics statement

The protocol for handling animal specimens in this study was approved by Universidad de San Francisco Xavier de Chuquisaca Animal Research Committee and follows the European Directive (Directive 2010/63/EU revising Directive 86/609/EEC on the protection of animals used for scientific purposes, project license number D.F.C.Q.F Y B. N° 237).

### Study area

**Figure 1:**
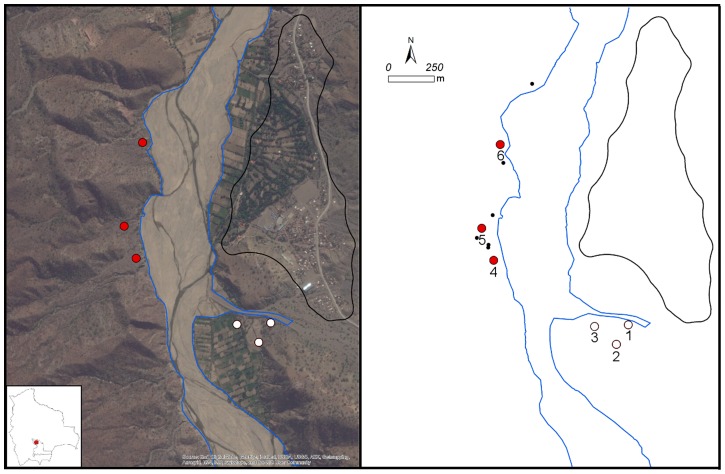
Satellite imagery (∼1 m resolution, ArcGIS, Ver. 10.1, ESRI Inc., Redlands, California, USA [26]) centered on the riverbed of Rio Chico near the town of Zurima (left). Digitized town boundary (black polygon), riverbed (blue lines), six isolated houses (small black dots) and numbered locations of the positive (red fill) and negative (white fill) trap sites.

Located in the Andean highlands of the department of Chuquisaca, Bolivia, the rural landscape of the study area includes xeric valleys [22], with thorny shrubs and cacti adapted to low, seasonal rainfall that defines the dry (8.4 cm average rainfall of May to October) and wet seasons (35.8 cm average rainfall of December to March) [23]. The sampling locations (Mean Center calculated at 65^o^ 08′ 2.31″ W, 18^o^ 46′ 44.26″ with ArcGIS, Ver. 10.1, ESRI Inc., Redlands, California, USA) were within 200 m of Rio Chico; six sampling locations were evenly divided between the west and east banks. Elevation of these locations range between 1740 m and 1780 m above mean sea level (AMSL, Figure 1).

The nearest community to the sampling locations is Zurima, ∼600 m to the east (Figure 1). Located along Rio Chico, Zurima has an elevation of 1730 m (Mean Center at 18^o^ 46′ 34.96″ S, 65^o^ 07′ 42.36″ W). As of the latest national census in 2001, Zurima had a population of 495 (257 females, 238 males) living in 135 houses [24]. The residents are mostly indigenous or of Spanish descent and practice subsistence agriculture [24], [25]. Six isolated houses, ranging 14 m to 870 m apart and ∼60 m from the closest trap location, are located on the west riverbank (Figure 1). These isolated houses were identified with satellite imagery but never surveyed.

### GIS analysis

The town boundary of Zurima was defined as a 50 m buffer from houses along the edge of the community. Six isolated houses were geolocated based on freely available satellite imagery (ArcGIS, Ver. 10.1, ESRI Inc., Redlands, California, USA) [26] (Figure 1).

A proximity function (Near) analysis was used to calculate the distance between the traps and town boundaries and thus, the distance of sylvan vectors from the town as well as the distance between the traps and isolated houses. ArcGIS was used to perform all GIS analyses (ArcGIS, Ver. 10.1, ESRI Inc., Redlands, California, USA).

### Insect vector collection

**Figure 2:**
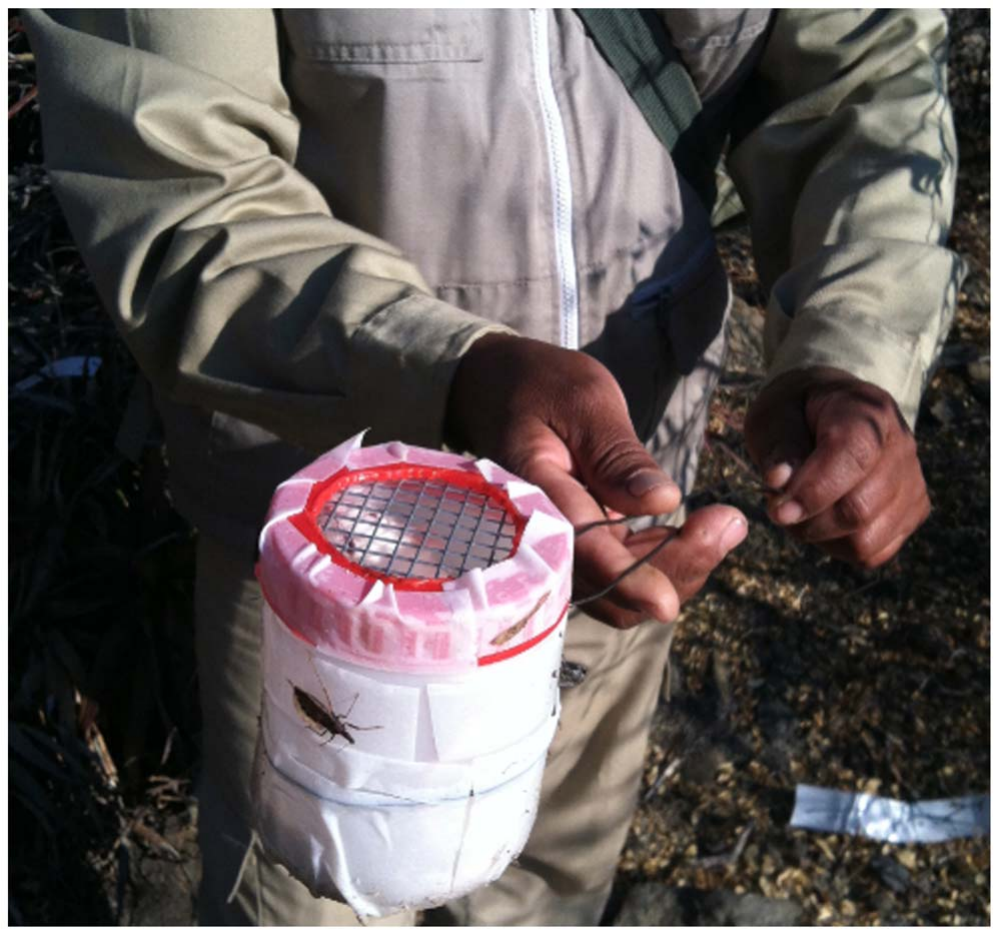
Noireau sylvatic insect vector trap. Traps consisted of plastic containers with a live mouse and wire mesh tops. Double sided sticky tape was placed on the outside of the traps.

Trapping many insect vectors is challenging due to the low success rates of traps [27]. We baited traps with live mice (also known as Noireau traps) because a review of the literature suggested these traps attract *T. infestans*, the principal insect vector in the region, more successfully than traps without mice [28] in laboratory studies, and that they have been successful in previous field studies [27], [29]. Traps consisted of opaque bottles (15×7 cm) covered with double-sided tape. A mouse was placed inside with a small piece of apple; and the opening was sealed with a metal screening mesh to prevent adult vectors and large nymphs from entering the trap (Figure 2). One to four traps were placed at six georeferenced sampling locations for a total of 17 traps (Figure 1). Traps west of the riverbed were placed in sylvan areas, while the eastern traps were between the village of Zurima and agricultural areas.

Traps were placed in the field at sunset on September 4^th^, 2012 at ∼18:00 and recovered ∼12 hours later. Vectors were gently removed from the double-sided tape with forceps, placed individually into plastic flasks, labeled according to the collection site, and transported live to the laboratory. Each trap location was recorded with a handheld GPS receiver (Garmin model 76, WGS 1984).

### Parasite detection and DNA extraction

*Trypanosoma cruzi* infection was determined using microscopy by USFX (Universidad San Francisco Xavier, Sucre, Bolivia) researchers trained in the safe handling of infectious agents using published methods [21]. DNA was subsequently extracted from the posterior abdomen of each insect using the DNeasy Kit (Qiagen, Valencia, CA) following the manufacturer's protocol, as previously described [10]. DNA concentrations of abdomen extractions were measured with the NanoDrop ND-100 spectrophotometer. These abdomen extractions, a complex mixture of degraded DNA containing parasite DNA (if infected), digested vector blood meal and vector tissue derived DNA, were also tested for *T. cruzi* by USFX researchers using PCR with the previously reported TCZ primers [21], [30]. PCR results were confirmed by SEDES (Servicio Departamental de Salud) in La Paz, Bolivia.

### Detection of blood meal sources

Blood meal sources were assayed using two methods: cloning of PCR products amplified with general vertebrate primers and qPCR using taxa specific primers and hydrolysis probes for chicken, *Canis sp.* and human blood meals (Table 1).

**Table 1:** Sequence of mitochondrial DNA primers (F  =  Forward, R  =  Reverse) and probes (P) used in cloning and qPCR assays to detect vertebrate blood meal sources in Chagas disease insect vectors.

Assay	Target DNA	Sequence (5′–3′)
Cloning	Vertebrate *12S* rRNA^1^	F: ACTGGGATTAGATACCCCACTATG
Melton		R: ATCGATTATAGAACAGGCTCCTC
Kitano	Vertebrate *12S* rRNA^2^	F: CCCAAACTGGGATTAGATACCC
		R: GTTTGCTGAAGATGGCGGTA
qPCR	Chicken Cyt *b* 3	F: TAGCCATGCACTACACAGCAGACA
		R: TTTGCGTGGAGATTCCGGATGAGT
		P: ACTTGCCGGAACGTACAATACGGCT
	*Canis sp*. Cyt b^3^	F: CCACAGCATTCATGGGCTATGTACT
		R: AGCTGCGATGATGAAAGGGAGGAT
		P: CAGTGGACAAAGCAACCCTAACACGA
	Human Cyt *b* 3	F: AGTCCCACCCTCACACGATTCTTT
		R: AGTAAGCCGAGGGCGTCTTTGATT
		P: ACCCTTCATTATTGCAGCCCTAGCAGCACT

1 [22].

2 [32].

3 [35].

### Detection of blood meal sources: Cloning

We analyzed the blood meal sources of all 14 vectors using the cloning assay following previously published methods [15], [31]. Briefly, because the DNA extractions from the insect abdomens potentially contain blood meal DNA from multiple vertebrates, the initial PCR used primers specific for mitochondrial DNA coding for the *12S* ribosomal RNA gene of vertebrates (hereafter referred to as *12S* primers). Two sets of vertebrate *12S* primers were used [22], [32], and are referred to hereafter as the Kitano and Melton assays. An ethidium bromide stained, 2% agarose gel was used to verify the ∼150 bp PCR products, which were then cloned with the pGEM-T kit (Promega, Madison, WI, USA). Cloned DNA from 12 colonies per insect (or 24 from two vectors because the first 12 did not have a single interpretable sequence) was PCR amplified using the same *12S* primers, sequenced using BigDye v3.1 (Applied Biosystems, Foster City, CA, USA) and subsequently analyzed with an ABI PRISM 3730*xl* DNA analyzer (Beckman Coulter, Fullerton, CA, USA). Sequence alignments and editing were done with Sequencher v4.10 (Gene Codes Corporation, Ann Arbor, MI, USA). Taxonomic identification of the sequences was determined as ≥99% match of 101 bp (Kitano) or 107 bp (Melton) using a BLAST search. To rule out contamination, two controls with nuclease free, DNA grade water instead of template DNA went through each step of the cloning and sequencing procedure for each set of primers (i.e., Melton and Kitano).

### Detection of blood meal sources: qPCR

Based on the results of the cloning assay, combined with previous studies analyzing blood meal sources of vectors from Bolivia, Brazil and Argentina [5], [6], [10], [33], [34], species-specific qPCR assays were developed to test for chicken, *Canis sp*. and human blood meal sources. The blood meal qPCR assays were modified from previously described assays targeting the mitochondrial cytochrome *b* gene (hereafter referred to as *Cyt*b, [35].

### Assay validation

The guidelines for the “Minimum Information for Publication of Quantitative qPCR Experiments” (MIQE) were followed to test the assays and interpret results [20]. We used the previously described [35] primer and probe concentrations for *Canis sp*. However, because the reported conditions for the chicken and human assays did not produce consistent results, we tested primer concentrations of 50–900 nM per reaction for chicken and human using DNA from known sources. After selecting the concentration yielding consistent amplification curves, we varied the probe concentration 50–900 nM. In addition, we varied annealing temperatures from 60°C to 62°C for the chicken and human assays. Following optimization, we analyzed the sensitivity of chicken, *Canis sp.* and human qPCR assays by varying the template DNA concentration using 10-fold serial dilutions spanning 10 orders of magnitude (10^0^ vs. 10^−10^) in triplicate. There were eight no template controls (NTC) consisting of DNA grade H_2_O instead of template. The 10^0^ template concentrations were 12.46 ng/uL of chicken DNA, 7.06 ng/uL of *Canis sp.* DNA and 7.90 ng/uL of human DNA.

After optimization and sensitivity analyses, chicken and human assays were run in duplicate including all samples, positive controls spanning five orders of magnitude (10^0^ to 10^−4^) and three or four NTC. We tested for *Canis sp.* in singlicate using half the amount of template used for the other qPCR assays because of limited DNA template. Because this is the first study to use hydrolysis probe-based qPCR to detect blood meal sources, the interpretation of the qPCR results of one replicate of each sample and at least five positive and three or four NTC controls, were verified by sequencing the qPCR products in one direction using the forward qPCR primer and the same sequencing protocol as the cloning assay. In three cases, because the duplicate trials differed, we sequenced both replicates. Although there was no evidence of NTC amplification before cycle 40, we sequenced NTC with the samples to rule out false positive results.

The qPCR reactions included 10 uL PerfeCTa qPCR ToughMix (Quanta Biosciences, Gaithersburg, MD, USA, Catalog # 95112-250), 8 uL of template DNA for human and chicken and 4 uL template DNA for *Canis sp.*, forward primer (200 nM chicken and *Canis sp*., 300 nM human), reverse primer (60 nM chicken, 200 nM *Canis sp*., 300 nM human), probe (230 nM chicken, 200 nM *Canis sp*., 300 nM human) and nuclease free water to make 20 uL. A two-step qPCR cycling protocol was used for all three assays. The chicken and human assays had an initial denaturation at 95°C for 10 minutes, followed by 45 cycles of 95°C for 20 seconds and 60°C for 1 minute, and a final extension of 72°C for 10 minutes. For the *Canis sp*. assay, the initial denaturation was for 5 minutes, followed by 45 cycles of 95°C for 5 seconds and 57°C for 40 seconds with final extension at 72°C for 10 minutes.

Both probes and primers were purchased from Biosearch Technologies (Novato, CA); and qPCR reactions were run on a LightCycler™ 480 thermocycler (Roche, Indianapolis, USA). Reagents and master mixes were prepared in a vented hood sterilized by UV light and wiped with 0.4% sodium hypochlorite to minimize contamination. Autoclaved tubes and DNA grade water, along with the DNA-free 96-well plates, filtered tips, pipettes and tube racks wiped with 0.4% sodium hypochlorite were placed 5–10 cm from a UV light source for 1 h before preparing all reagents and master mixes [36].

To compare sylvatic vector blood meal feeding samples to the positive and negative controls, the crossing point (Cp), defined as the cycle where the sample begins to amplify above the background noise [20], [37], [38], was calculated using the Absolute Quantification Fit Points method from the LightCycler 480 GeneScanner Software V1.5 (Roche, Indianapolis, USA). Reactions were run for 45 cycles. The 40 cycle threshold represents the cycle where significant amplification should be visible if there is at least one molecule of target DNA in the original sample [37].

## Results

### Insect vector collection and parasite infection

Chagas disease vectors were collected at three of the six sampling locations, located furthest from Zurima, and from five of the 17 (30%) traps. All 14 vectors were collected within 100 m of an isolated house (Table S1); and all were *T. guasayana*, a species that has been previously reported in domestic [2], peridomestic and sylvatic [39] ecotopes. The concentration of extracted DNA averaged 33.3 ng/uL (range 2.4–175.22 ng/uL). *T.cruzi* was detected in only one vector (7%) with complete agreement between microscopy and PCR.

### Cloning blood meal detection

The initial PCR products from the 14 *T. guasayana* for both the Melton and Kitano assays were the expected size and thus used in the cloning reactions; even though there was no visible band from the negative control, it was treated the same as the samples to control for potential contamination. The PCR products from the cloning colonies of all 14 samples were also the expected size; the colonies from the negative control did not produce the expected band size indicating there was no contamination. For the Melton assay, 12 colonies from each sample were sequenced. Because no interpretable sequences were obtained from the first 12, an additional 12 colonies were sequenced for samples Tg04 and Tg11. For the Kitano assay, 12 colonies from each sample were sequenced.

Of the 14 *T. guasayana* analyzed, four different blood meal sources were detected with the Melton cloning assay; three of these sources were found in more than one *T. guasayana* (Table 2). The most common blood meal source was human, which was found in six *T. guasayana*. Five *T. guasayana* had evidence of feeding on chicken (*Gallus gallus*) and five had fed on unicolored blackbird (*Agelasticus cyanopus*). Opossum (*Monodelphis domestica*) was detected in one *T. guasayana*. The Melton cloning assay detected 57% (8 of 14) of *T. guasayana* feeding on birds from both domestic and sylvatic ecotopes, and 14.3% (2 of the 14) of the vectors feeding on birds were positive for both chicken and unicolored blackbird. Based on the Melton cloning assay, 50% (6 of 14) fed exclusively on domesticated taxa; 14.3% (2 of 14) fed exclusively on sylvatic taxa (i.e., unicolored blackbird); 28.6% (4 of 14) fed on vertebrates from both domestic and sylvatic ecotopes. We were unable to detect a blood meal source for 7% (1 of 14) vectors despite analyzing 24 colonies instead of 12.

**Table 2:** Blood meal sources of the Chagas disease vector, *Triatoma gusayana* collected from sylvatic locations in Bolivia.

ID-trap site	Vector stage or sex	*T. cruzi* infection	No. taxa detected by cloning	Human cloning	Human qPCR	Chicken cloning	Chicken qPCR	Dog cloning	Dog qPCR	Blackbird cloning	Opossum cloning
Tg01-4	4 or 5	-	1		+	+	+		+		
Tg02-4	4	-	1		+	+	+				
Tg03-4	2	-	2		+	+	+			+	
Tg04-4	3	-	0		+		+		+		
Tg05-4	F	-	1	+	+		+		+		
Tg06-4	F	-	2	+	+		+		+	+	
Tg07-4	M	-	1		+		+				+
Tg08-4	M	-	3	+	+	+	+			+	
Tg09-4	3	-	1	+	+		+				
Tg10-5	F	-	1		+		+			+	
Tg11-5	2	-	1	+*	+		+				
Tg12-5	M	-	2	+	+	+	+				
Tg13-6	F	-	1		+					+	
Tg14-6	F	+	1	+	+		+				
Average or % with blood meal source			1.36	50%	50–100%+	36%	93%	0%	29%	36%	7%

Blood meal sources were determined by either a cloning assay or qPCR using DNA extracted from the vector abdomen.

* Detected by Kitano cloning assay, all other blood meals identified by cloning were detected with the Melton assay.

+ See text for discussion of human qPCR assay.

For the Kitano cloning assay, only one blood meal source, human, was detected, and from only one vector (Table 2). Overall, the Kitano primers were less successful than the Melton primers, the Kitano assay blood meals were detected from 7% vs. 86% of samples for the Melton primers. Although both authors claim the primers are for species identification among vertebrates, a comparison of sequences in this region of the *12S* gene shows that the match varies among taxa and for the Kitano primers, appears to be higher in mammals than birds (Table 3). Interestingly, the Melton primer set did not detect the same blood meal source as the one blood meal the Kitano primer set detected.

**Table 3:** Match between primers used in the cloning assays to identify blood meal sources of *Triatoma guasayana* and the taxa detected, showing that the Melton primers are a better match than the Kitano primers.

taxa	*12S* Forward (5′ to 3′)	*12S* Reverse (5′ to 3′)
Melton *12S*	ACTGGGATTAGATACCCCACTATG	ATCGATTATAGAACAGGCTCCTC
Chicken^1^	------------------------	-----------------------
Blackbird^2^	------------------------	-----------------------
Human^3^	------------------------	--------C--------------
Opossum^4^	------------------------	-----------------------
Dog^5^	------------------------	-----------------------
Kitano *12S*	CCCAAACTGGGATTAGATACCC	GTTTGCTGAAGATGGCGGTA
Chicken^1^	----------------------	—CC----CG—G-------
Blackbird^2^	----------------------	—CC----CG—G-------
Human^3^	----------------------	--------------------
Opossum^4^	TA--------------------	—CC----G-----------
Dog^5^	----------------------	--------------------

1 Chicken (*Gallus gallus*), GenBank accession number HQ857212.1.

2 Unicolored blackbird (*Chrysomus cyanopus*) JX516076.1.

3 Humans (*Homo sapiens*) NC_012920.1.

4 Opossum (*Monodelphis domestica*) AJ508398.1.

5 Dog (Canis lupus familiaris) JF342907.

Although dogs are a common blood meal source in this region, they were not detected as a blood meal source by either of the cloning assays. The cloning NTC in duplicate, for both the Melton and Kitano assays, did not produce interpretable DNA sequence, ruling out contamination in the cloning assays.

### QPCR blood meal detection

**Figure 3:**
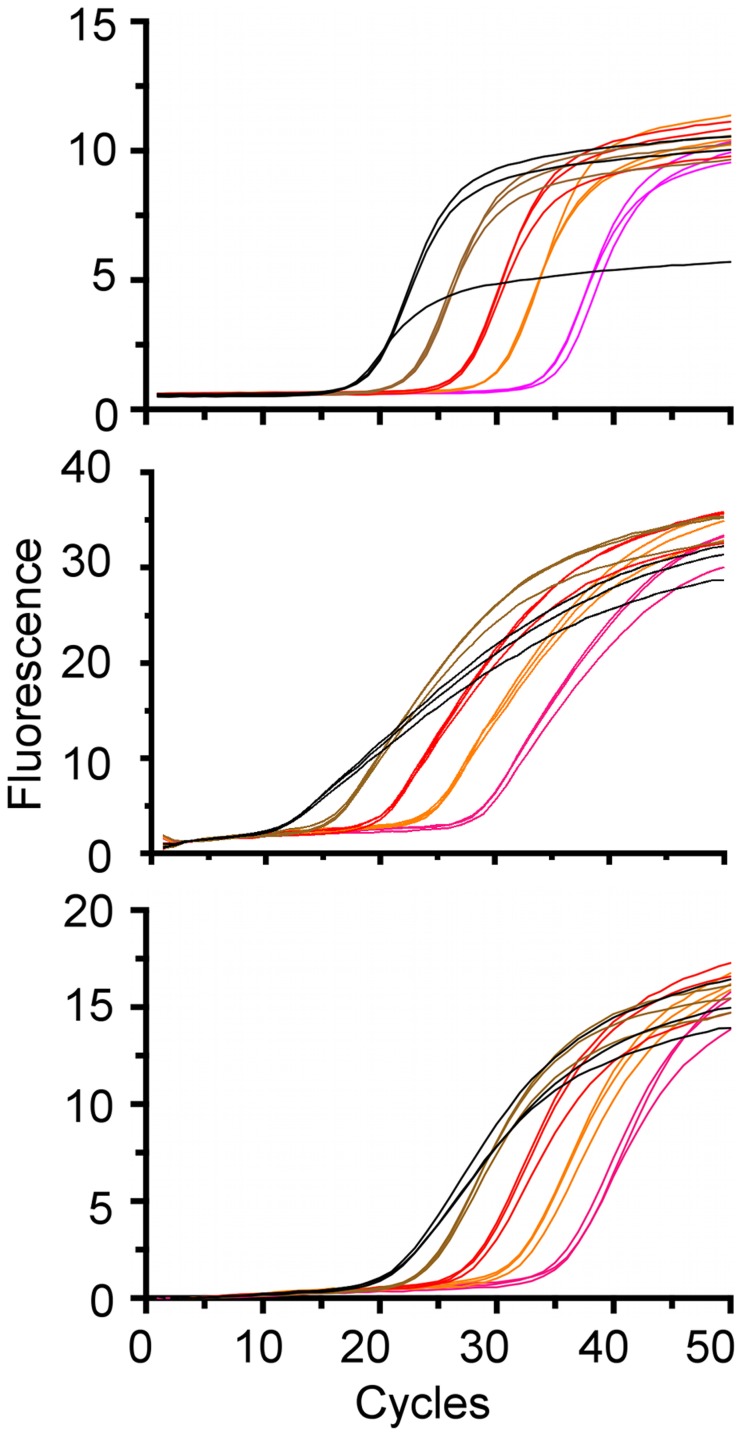
Fluorescence as a function of reaction cycles for (A) chicken, (B) *Canis sp.* and (C) human. Positive controls were run in triplicate starting with 10^0^ on the left (black) followed by each successive 10-fold serial dilution in a different color. Amplification curves are shown for the dilutions above the 10^−4^ limit at which template concentration that can be detected with 95% certainty.

**Figure 4:**
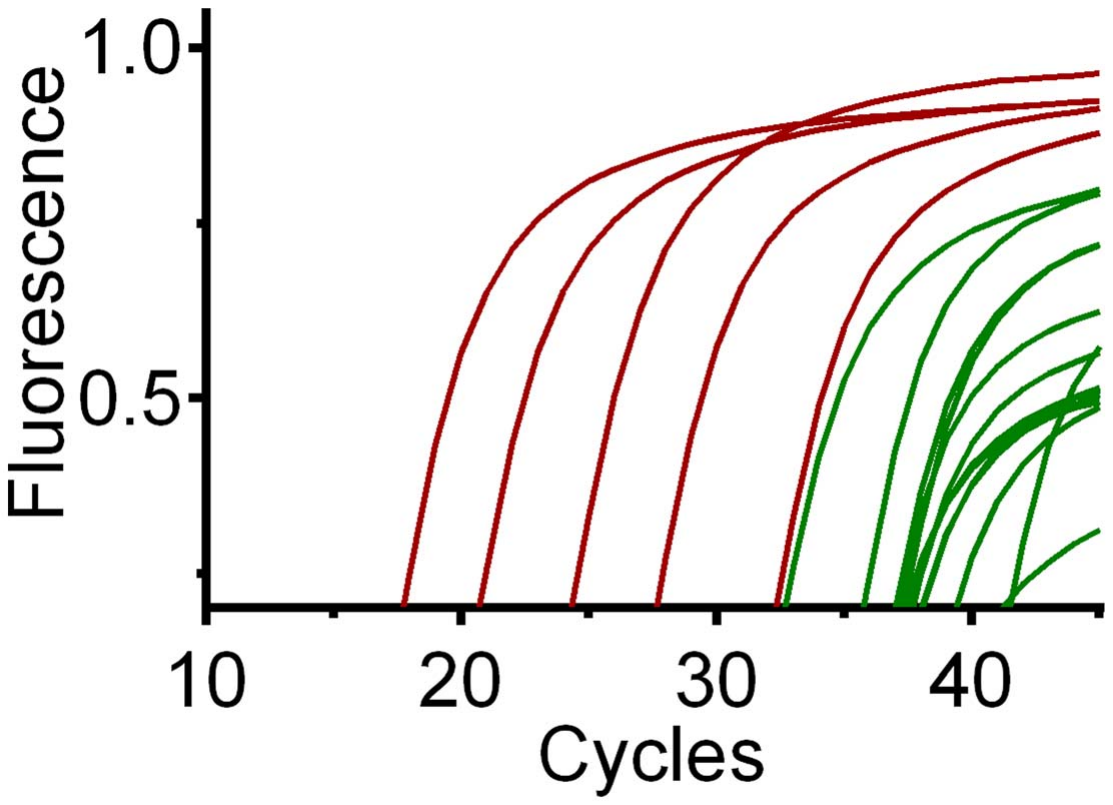
Chicken qPCR amplification of 14 samples (green), 5 serial dilutions (red) and NTC (no template control, black). NTC are not visible because they did not amplify and rise above the X-axis.

**Figure 5:**
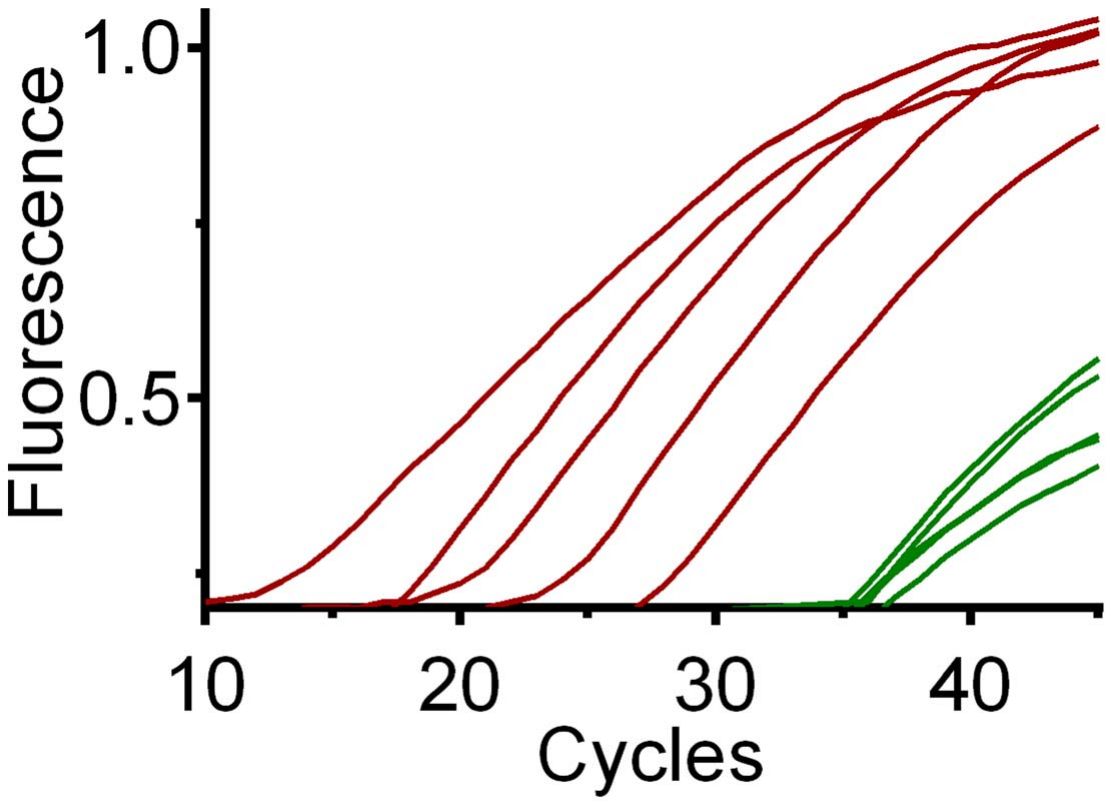
*Canis sp.* qPCR amplification of 14 samples (green), 5 serial dilutions (red) and NTC (no template control, black). NTC are not visible because they did not amplify and rise above the X-axis.

**Figure 6:**
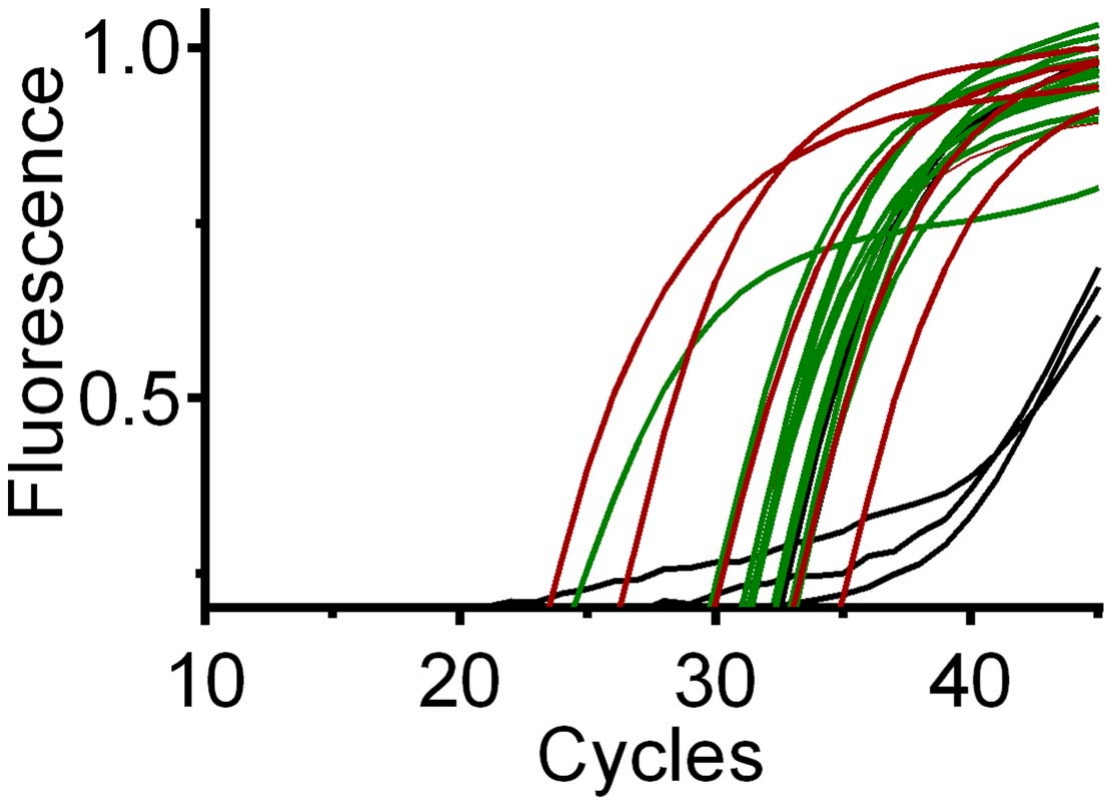
Human qPCR amplification of 14 samples (green), 5 serial dilutions (red) and NTC (no template control, black).

**Figure 7:**
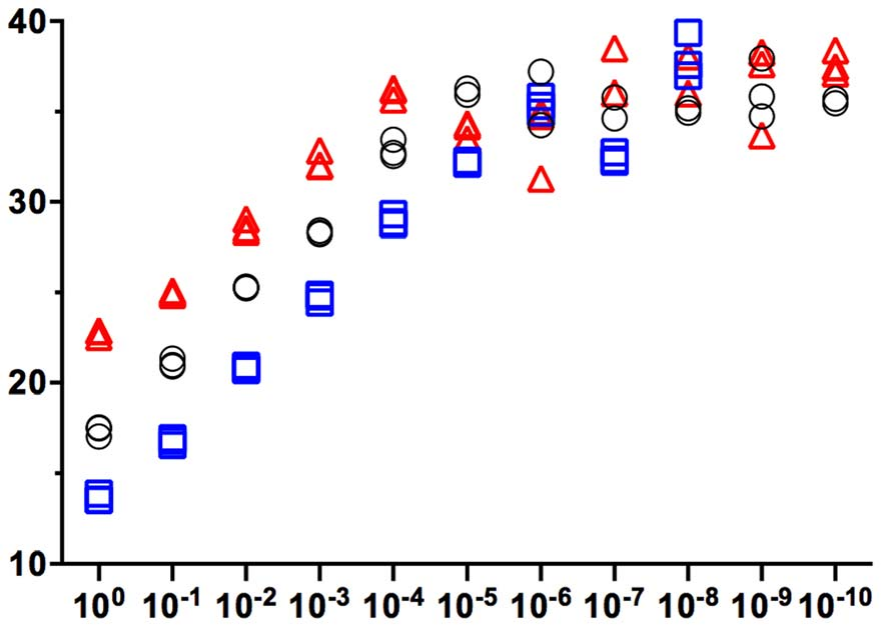
Cp (crossing point) vs. serial dilutions for chicken (black circle), *Canis sp.* (blue square) and human (red triangle) assays in triplicate.

The limit of detection (LOD), or template concentration that can be detected within 95% certainty [20], is 10^−4^ ng/uL for all three qPCR assays (Figure 3). Although positive controls were detected at more dilute concentrations, we could only yield consistent and reproducible results until 10^−4^ ng/uL. Averages of three replicates per concentration are 3.9 (range 3.7–4.6) cycles apart for chicken (Figure 4), 3.8 (range 3.1–4.3) cycles apart for *Canis sp.* (Figure 5) and 3.3 cycles (range 2.2–3.8) apart for human (Figure 6), respectively, a little higher than the expected 3.3 cycles [37]. The more dilute samples (<10^−5^ ng/uL) sometimes amplified after the 40 cycles at which one DNA molecule should be detectable (Figure 7) [37]. Four to five positive controls for chicken, *Canis sp.* and human qPCR products were sequenced, including the 10^−4^ ng/uL sample, and correctly identified in a BLAST query.

For the qPCR assay, we found the prevalence of feeding on *Canis sp.*, chicken and human was 29%, 93%, and 50 or 100% (4, 13 and 7 or 14 of 14) respectively (results summarized in Table 2, Cp values for each sample in each assay are shown in Table S2). DNA sequencing confirmed the amplicon was the target DNA in all instances. Attempts to sequence the qPCR product from the NTC were unsuccessful for the chicken and *Canis sp.* assays. In contrast, two of the four human NTC had amplicons identified as human by BLAST search; however, the NTC amplified five cycles later than the lowest positive control (10^−4^ ng/uL, LOD) and six cycles later than the latest amplifying sample (Table S2). Because of the human DNA amplification from the NTC, we conservatively conclude that at least 50% (the number positive by the cloning assay) of the samples are positive for human. Note that the samples positive for human by cloning tend to have the lowest Cp values, indicating higher concentrations of DNA (samples positive for human by cloning average: Cp  = 27.6, average for samples negative for human by cloning: Cp  = 34.0).

### Comparison of blood meal detection methods

The qPCR assay detected its targeted taxa (i.e., chicken, *Canis sp.* and human) in more blood meals than the cloning assay. For chicken, all five samples identified as positive by the cloning assay were also positive by qPCR; however, qPCR showed that an additional sample had fed on chicken. Similarly for human, all seven samples that were positive by the cloning assays were positive by qPCR and qPCR indicated that the remaining seven were positive for human; however, due to reported NTC amplification (see **QPCR blood meal detection**), only the human cloning results can be unambiguously established. Although the cloning did not detect any feeding on *Canis sp*., the qPCR assayed was positive for four samples.

## Discussion

We collected 14 sylvatic *T. guasayana* during the dry season (September) in the Andean highlands of the department of Chuquisaca, Bolivia and found a single individual positive for *T. cruzi* by both microscopy and PCR. Using the cloning assay and our newly developed, highly sensitive qPCR assay, we also found at least 50% were positive for human blood meal indicating *T. guasayana* should be further examined for potential epidemiological importance because our study did not explicitly examine parasite transmission.

Our study reports on a limited number of *T. guasayana* because cloning is very resource intensive (see **Cloning blood meal detection**); however, additional specimens should be examined for a more complete picture of sylvatic *T. guasayana* feeding habits. *T. guasayana* from northern Argentina have been reported previously to have a high rate of feeding on humans (3 of 5 blood meals analyzed against blood antisera) and carry a *T. cruzi*-like parasite (2 of 5 analyzed with microscopy), although none of the vectors were positive for both human and the parasite [6], [40].

We detected, on average, 1.4 blood meal sources per vector using the cloning assays. Evidence of domestic (chicken, *Canis sp.* and human) blood meal sources was found in all vectors with qPCR. Literature reports adult *T. guasayana* as having high night flight dispersal during the dry season [27], [40]. Previous studies of sylvatic Chagas disease vectors in Argentina found the dispersion index for *T. guayasana* was 4.5 times higher for females and 2 times higher for males compared to *T. infestans* the most prevalent domestic vector in this region [41]. They found *T. guasayana* had the highest number of flying individuals and although it does not colonize houses, adults often invade houses. Based on our trap locations, these vectors appear highly mobile, travelling between ∼60 m (nearest isolated house) and ∼600 m (nearest house in Zurima) to obtain blood meals from domestic sources. The collection sites for *T. guasayana* from this study are 60 m. (Table S1) to the nearest isolated house, which is within the 80 m nymph dispersal distance reported in a study from Argentina. Thus previous studies indicate it is plausible for *T. guasayana* to move to houses, however the food sources also move. Evidence (e.g. trash, fire pits, hunting remains) of humans sleeping/resting in sylvan areas has been reported [42], [43] and chickens often roam freely [44], so this result could be from either vectors or hosts moving, or more likely, a combination of both.

All 14 *T. guasayana* from this study had evidence of feeding on domestic animals, but about half also fed on sylvatic animals (unicolored blackbird and opossum). Sylvatic *T. guasayana* from Argentina in previous studies have been reported to exclusively feed on domestic hosts based on an antisera test relying on controls gathered from local animals [45]. The majority of controls used in this Argentinian study were animals not typically found in sylvan areas (72.7%, 8 of 11) [6], [45]. Our cloning method reported here does not rely on the availability of, nor is it limited by *a priori* knowledge of domestic, peridomestic and sylvatic fauna for testing blood meals.

Although qPCR indicates *T. guasayana* in our 14 samples are not feeding on *Canis sp.* as frequently as humans and chickens, dogs have been suggested as sentinels of *T. cruzi* infection in humans [46]. A rural town in northern Argentina reported as many as 65% of the dogs seropositive for *T. cruzi* infection [46]; and *T. guasayana* was correlated with the *T. cruzi* infection of the dog population [46].

The incidence of *T. cruzi* in sylvatic hosts can give some insight into the importance of this broad feeding on the potential transmission of *T. cruzi* to humans. Sylvatic rodents have been reported as blood meal sources for sylvatic vectors in Central [18] and South America [17] and are an important *T. cruzi* reservoir with reportedly high parasitemia [47]; likewise, opossum are an important reservoir for *T. cruzi* throughout its range [48]. Although birds cannot sustain *T. cruzi* infection, having chickens near domiciliary areas may decrease vector parasitemia, while increasing vector abundance [7].

Fluorescence based qPCR assays are capable of detecting minute amounts of nucleic acids while being quick, simple and specific [20]. Of the two basic qPCR approaches (i.e., high resolution melting (HRM) and hydrolysis probe assays), HRM assays have recently been reported for detecting multiple blood meals; however, the resolution of taxa with similar Tm [11] (for example chicken and human 86.27 and 85.79, respectively) could yield indistinguishable genotype signatures. Similarly, a recent study used HRM to detect blood meals using *12S* vertebrate primers; however, multiple feedings by a vector was not examined [12]. This is the first report of a hydrolysis probe-based qPCR assay for detecting vector blood meal sources. Probe-based assays can be multiplexed and have the potential to be more specific, more sensitive, and more reproducible. Although the assays reported here examined each taxa in a separate assay, multiplex assays could be developed using probes with different fluorescence for each potential vertebrate host.

There are few controlled experiments for blood meal detection. Although insect vectors require a feeding to molt into each lifecycle stage and many Triatominae can live up to two years [49], [50], researchers have detected blood meals in lower abdomen extractions only 70 days post feeding using less sensitive PCR methods [18]. Numerous studies have used days post feeding as an indicator of assay sensitivity [9], [11], [18], [19]. An alternative approach is to assess PCR sensitivity with serial dilutions, while examining any temporal effects of blood meal digestion using days post feeding. One study successfully detected template until 21 days post feeding with sensitivity reported until 10^−1^, 10^−3^ and 10^−2^ for chicken, dog and human, respectively [51]. Our study is the first to examine qPCR sensitivity using serially diluted controls. We found a lower detection limit and thus present more sensitive assays than reported for conventional end-point PCR (chicken: 3 orders of magnitude, *Canis sp.*: 1 order of magnitude and human: 2 orders of magnitude) [51]. Temporal effects of blood meal digestion has not yet been examined for hydrolysis probe-based qPCR.

Despite extreme efforts to control for contamination, the qPCR assay showed amplification of human DNA in the NTC, suggesting caution in interpreting results. We ran samples in duplicate or triplicate with high volumes of template DNA to reduce the likelihood of false positives. Sequencing of qPCR products, used to verify results, showed we were unable to eliminate human contamination; however, the combination of the cloning assay results and the qPCR amplification of samples at least 5 cycles before the NTC supports our conclusion that a significant number of these vectors are feeding on humans. Previous studies have shown some reagents may be contaminated with human DNA and have encouraged researchers to position tubes on their sides with UV treatment within 2.54 cm [52], [53], differing from our upright placement of tubes with DNA grade water within 10 cm of a UV light source. Therefore we speculate our DNA grade water, used to prepare other reagents, as a potential source of contamination.

This study demonstrates the power of combining the cloning assay with the highly sensitive hydrolysis probe-based qPCR to provide a more complete picture of the blood meal sources of insect disease vectors. Our data show that humans and chickens are major food sources for sylvatic *T. guasayana* based on the qPCR assay, while the cloning assay was able to discover that *T. guasayana* are also feeding on wild animals. Future studies should include sensitivity analysis of qPCR after blood meals have been subject to various lengths of digestion by the vector and, in addition, would benefit from this dual approach, the cloning assay to gather *a priori* information on actual food sources perhaps on a larger sample size of pooled triatomine blood meals, followed by the highly sensitive qPCR assay to determine feeding habits of individual triatomines. Combined with Noireau traps, these methods can help us unravel the role of sylvatic insect vectors in the transmission of human Chagas disease.

## Supporting Information

Table S1 Summary of trap sites and isolated houses.
(DOCX)

Table S2 Summary of qPCR and cloning blood meal assays of sylvatic *Triatoma guasayana*, showing that taxa identified by cloning were also always detected by qPCR; however, taxa identified by qPCR were not always identified by cloning. The Cp (crossing point) and sequence ID of the qPCR product or cloned DNA are shown; sequence IDs are based on 100% match in a BLAST search of>100 nucleotides.
(DOCX)
