# Supplementary material for: Sources of Blood Meals of Sylvatic Triatoma guasayana near Zurima, Bolivia, Assayed with qPCR and 12S Cloning
Source: PLoS Negl Trop Dis. 2014 Dec 4;8(12):e3365. doi: 10.1371/journal.pntd.0003365 (PMC4256209; doi:10.1371/journal.pntd.0003365)
Supplement: Table S1 — Summary of trap sites and isolated houses. (DOCX) [file pntd.0003365.s001.docx]

Table S1 Summary of trap sites and isolated houses.

| Trap Site | No. of Adults | No. of Nymphs | Trap Site Location | Nearest Isolated House (m) | Distance to Zurima Boundary  (m) |
| --- | --- | --- | --- | --- | --- |
| 1 | 0 | 0 | 65°8'11.09"W 18°46'23.03"S | 804.6 | 515.6 |
| 2 | 0 | 0 | 65°8'14.60"W 18°46'37.11"S | 806.6 | 509.5 |
| 3 | 0 | 0 | 65°8'12.61"W 18°46'42.03"S | 664.9 | 446.6 |
| 4 | 4 | 5 | 65°8'12.51"W 18°46'42.08"S | 59.3 | 347.1 |
| 5 | 2 | 1 | 65°8'14.51"W 18°46'36.81"S | 65.9 | 327.7 |
| 6 | 2 | 0 | 65°8'11.09"W 18°46'23.08"S | 97.3 | 227.1 |
