# Supplementary material for: Sources of Blood Meals of Sylvatic Triatoma guasayana near Zurima, Bolivia, Assayed with qPCR and 12S Cloning
Source: PLoS Negl Trop Dis. 2014 Dec 4;8(12):e3365. doi: 10.1371/journal.pntd.0003365 (PMC4256209; doi:10.1371/journal.pntd.0003365)
Supplement: Table S2 — Summary of qPCR and cloning blood meal assays of sylvatic Triatoma guasayana, showing that taxa identified by cloning were also always detected by qPCR; however, taxa identified by qPCR were not always identified by cloning. The Cp (crossing point) and sequence ID of the qPCR product or cloned DNA are shown; sequence IDs are based on 100% match in a BLAST search of>100 nucleotides. (DOCX) [file pntd.0003365.s002.docx]

Table S2 Summary of qPCR and cloning blood meal assays of sylvatic *Triatoma guasayana*, showing that taxa identified by cloning were also always detected by qPCR; however, taxa identified by qPCR were not always identified by cloning. The Cp (crossing point) and sequence ID of the qPCR product or cloned DNA are shown; sequence IDs are based on 100% match in a BLAST search of >100 nucleotides.

| Taxa | Chicken . | | | | | Dog . | | | Human . | | | | | |
| --- | --- | --- | --- | --- | --- | --- | --- | --- | --- | --- | --- | --- | --- | --- |
| Assay | qPCR . | | | | cloning | qPCR . | | cloning | | qPCR . | | | cloning |  |
| ID | Cp 1 | Cp 2 | sequence | sequence | | Cp | sequence | sequence | | Cp 1 | Cp 2 | sequence | sequence |  |
| Tg01 | 35.34 | 34.92 | Chicken | Chicken | | 40.08 | Dog | - | | 34.78 | 34.64 | Human | - |  |
| Tg02 | 34.96 | 35.62 | Chicken | Chicken | | - | - | - | | 34.97 | 35.26 | Human | - |  |
| Tg03 | 34.7 | 35.49 | Chicken | Chicken | | - | - | - | | 32.87 | 33.84 | Human | - |  |
| Tg04 | 33.37 | 37.67 | Chicken | - | | 41.23 | Dog | - | | 34.64 | 34.67 | Human | - |  |
| Tg05 | 36.84 | 34.71 | Chicken | - | | 41.7 | Dog | - | | 27.46 | 27.66 | Human | Human |  |
| Tg06 | 34.48 | 36.53 | Chicken | - | | 39.34 | Dog | - | | 32.83 | 33.38 | Human | Human |  |
| Tg07 | 35.3 | 34.63 | Chicken | - | | - | - | - | | 33.1 | 32.77 | Human | - |  |
| Tg08 | 34.69 | 35.88 | Chicken | Chicken | | - | - | - | | 34.03 | 33.94 | Human | Human |  |
| Tg09 | - | 30.22 | -; Chicken | - | | - | - | - | | 33.23 | 33.33 | Human | Human |  |
| Tg10 | 34.48 | 34.86 | Chicken | - | | - | - | - | | 32.87 | 33.1 | Human | - |  |
| Tg11 | - | 36.44 | -; Chicken | - | | - | - | - | | 33.85 | 33.93 | Human | Human |  |
| Tg12 | 38.85 | - | Chicken; - | Chicken | | - | - | - | | 31.7 | 31.43 | Human | Human |  |
| Tg13 | - | - | - | - | | - | - | - | | 35 | 35.32 | Human |  |  |
| Tg14 | 34.63 | 35.34 | Chicken | - | | - | - | - | | 34.32 | 34.15 | Human | Human |  |
| +C 10^-4^ | 29.75 | 29.68 | 64 | - | | 31.5 | 149 | - | | 36.86 | 36.9 | Human | - |  |
| NTC | - | - | - | - | | - | - | - | | 42.17 | 42.49 | Human* |  |  |

* Two of four samples were positive for human.
